# Listening to the rumours: What the northern Nigeria polio vaccine boycott can tell us ten years on

**DOI:** 10.1080/17441692.2013.859720

**Published:** 2013-12-03

**Authors:** Isaac Ghinai, Chris Willott, Ibrahim Dadari, Heidi J. Larson

**Affiliations:** a Institute for Global Health, University College London, London, UK; b Department of Infectious Disease Epidemiology, London School of Hygiene and Tropical Medicine, London, UK; c Clinton Health Access Initiative, Abuja, Nigeria

**Keywords:** polio, eradication, Nigeria, public trust, vaccine confidence

## Abstract

In 2003 five northern Nigerian states boycotted the oral polio vaccine due to fears that it was unsafe. Though the international responses have been scrutinised in the literature, this paper argues that lessons still need to be learnt from the boycott: that the origins and continuation of the boycott were due to specific local factors. We focus mainly on Kano state, which initiated the boycotts and continued to reject immunisations for the longest period, to provide a focused analysis of the internal dynamics and complex multifaceted causes of the boycott. We argue that the delay in resolving the year-long boycott was largely due to the spread of rumours at local levels, which were intensified by the outspoken involvement of high-profile individuals whose views were misunderstood or underestimated. We use sociological concepts to analyse why these men gained influence amongst northern Nigerian communities. This study has implications on contemporary policy: refusals still challenge the Global Polio Eradication Initiative; and polio remains endemic to Nigeria (Nigeria accounted for over half of global cases in 2012). This paper sheds light on how this problem may be tackled with the ultimate aim of vaccinating more children and eradicating polio.

## Introduction

In 1988 the World Health Assembly launched a campaign to eradicate polio by 2000. The Global Polio Eradication Initiative (GPEI) is a joint effort between the World Health Organization (WHO), United Nations Children's Fund (UNICEF), Rotary International, US Centres for Disease Control (CDC) and national governments. The partnership achieved phenomenal success: global incidence fell from over 350,000 in 1988 to just under 500 cases of polio-induced paralysis in 2001 (GPEI, 2013), but since then, successes have been much harder won with stubborn endemic pockets remaining.

The year 2012 marked major progress for GPEI. Just 223 cases were recorded compared to 650 the year before. India, once considered the most difficult eradication terrain, has now celebrated more than two polio-free years, breaking a chain of transmission that has lasted for centuries.

Despite this, a number of challenges face the initiative: waning political will and funding shortfalls (Aylward & Tangerman, 2011); occasional outbreaks – such as the on-going outbreak in the Horn of Africa (GPEI, 2013); continuing security and service delivery challenges (Closser, 2010); and risks associated with a live vaccine (Heymann, Sutter, & Aylward, 2006; Nathanson & Fine, 2002). Amidst these, public acceptance of vaccines remains important (Larson & Ghinai, 2011; Taylor & Shimp, 2010). Rumours associating oral polio vaccine (OPV) with fears of cancer, HIV and sterility occasionally flare causing sporadic cases of vaccine refusals and polio outbreaks (Bego, 2003; Thacker & Shendurnikar, 2004; UNICEF, 2001, 2012; Walsh, 2007).

A particularly serious and well-documented set of refusals occurred in Nigeria. Between July 2003 and August 2004, five northern Nigerian states suspended the use of OPV. Zamfara, Kaduna, Bauchi and Niger states rejoined National Immunisation Days within a few months but Kano state authorities did not allow vaccination to resume until a year later (IRIN, 2004).

The boycotts proved a huge setback for polio eradication. Incidence in Nigeria jumped from 202 in 2002 to 1143 in 2006 and Nigerian strains of the virus spread across Africa and beyond (Aylward & Heymann, 2005). Outbreak response activities cost the GPEI over $500 million (Kaufmann & Feldbaum, 2009).

**Figure 1.**
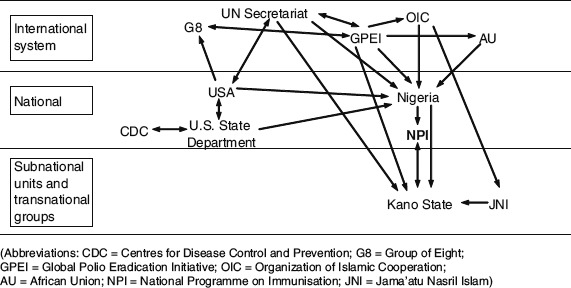
Interactions between global actors working to resume polio eradication in Kano State. Source: Kaufman & Feldbaum, 2009. Note: Notice the relatively disaggregated ‘Subnational units’.

The boycotts ostensibly came about in response to rumours, endorsed by high-ranking public figures, that OPV was an American conspiracy to spread HIV and cause infertility in Muslim girls (Raufu, 2002). However, deeper analyses indicate more complex factors were at play. The international response to these boycotts was impressive and successful negotiations eventually restored immunisation in each state (Figure 1, Kaufmann & Feldbaum, 2009). Whilst Figure 1 depicts the complex web of global political responses to the boycott, analysis of subnational activity remains relatively undifferentiated. Kimmel (2004) argues that ‘success in controlling a rumour, as when one attempts to fight a medical disease or illness, rests in the correct diagnosis of a rumour's nature’. We argue that the internal dynamics of the boycott have not yet received sufficient scrutiny. We therefore analyse societal factors that enabled these rumours to catch on and spread at the grass-roots level and identify the important views of several key individuals who fuelled the issue. We argue that the complexity of society, politics and personalities in Kano warrants further study, and that lessons can still be learnt from the local context – lessons that may be applied today.

## Methodology

Data for this analysis were collected from peer reviewed material and grey literature. The literature review was supplemented with interviews with key informants.

A body of published literature has investigated the political determinants of the Nigerian boycott. Grey literature was also searched: conference presentations, reports from national and international agencies, media reports, online sources and unpublished essays. Media articles and reports were found through targeted searching of regional sites (e.g. allAfrica.com) and passive collection using online media surveillance through the Vaccine Confidence Project (for a detailed methodology of the Vaccine Confidence Project, see Larson et al., 2013). We did not discriminate between factual sources or those published to propagate an ideology (such as material distributed by anti-vaccination groups); each were seen as contributing to the social fabric that allowed the rumour to flourish. Key themes were elucidated from the literature and investigated in historical and anthropological depth through subsequent literature searches.

Interviews were conducted with several key stakeholders to supplement the analysis. Professor David Heymann (former WHO Special Representative for Polio Eradication), Sebastian Taylor (former UNICEF/WHO consultant for polio eradication), WHO officials in Geneva (who wish to remain anonymous) and Professor Haruna Kaita (Dean of the Faculty of Pharmaceutical Sciences at Ahmadu Bello University, Zaria, Nigeria) were consulted.

## Societal factors

### Religion and ethnicity

One of the justifications given for the boycott was the belief that OPV spread HIV and caused sterility in Muslim girls. An understanding of the religious elements at play, which, in northern Nigeria, are intrinsically interwoven with ethnic identity, is therefore key to understanding the power this accusation had.

The north of Nigeria is home to a Muslim majority. Hausa and Fulani – the main ethnic groups in the north – are culturally distinct from the predominantly Christian population groups in the south (Nnoli, 1978), and Islam is an integral part of their identity (Last, 2008). Last (2008, p. 43) argues that the incorporation of independent Islamic regions into a secular, unified Nigeria in 1914 left a ‘pervasive anxiety’ among Muslims in the north reinforced by divisive colonial policies (Mustapha, 2002; Ukiwo, 2003). An ‘inter-ethnic rivalry for domination’ (Afigbo, 1989) still permeates many public debates.

Tensions between northern states and the Federal government flared in 2002 – one year before the OPV boycott – stirred by a religious issue. Sharia law, practiced in all states that boycotted immunisations, was deemed ‘unconstitutional’ by the Federal government of President Olusegun Obasanjo – a Yoruba, born-again Christian from south-western Nigeria. The president of the Supreme Council for Sharia in Nigeria (SCSN), Dr Datti Ahmed, claimed Muslims would go to war over this issue if challenged (Harnischfeger, 2004). Dr Ahmed and the SCSN would play a crucial role in the OPV boycott one year later.

Animosity was also building between some Islamic states and Western powers around 2003. The 2001 US-led invasion of Afghanistan and the war in Iraq (which began in March 2003, just three months before the boycott) fuelled an impression that there was a religious crusade by Christian, Western countries again Islam. Medicine was viewed as a vehicle of this geopolitical crusade: one northern Nigerian commented, ‘If America is fighting people in the Middle East, the conclusion is that they are fighting Muslims’ (Murphy, 2004).

This perceived threat to Islam had direct repercussions for the rumours around OPV: psychoanalytic approaches explain that creating common enemies through rumours can serve as a defence mechanism where community values are threatened (Kimmel, 2004). In northern Nigeria in 2003, a threatened community united around the ‘common enemy’ of Western interventions. As a result, some saw ‘opposition to polio vaccination as a means of expressing their anti-Western feelings’ (Yahya, 2007, p. 191). Sule Ya'u Sule, then Kano state spokesman, summed up the association: ‘Since September 11, the Muslim world is beginning to be suspicious of any move from the Western world. Our people have become really concerned about polio vaccine’ (News 24, 2004, p. 1).

Local perceptions of polio were also in direct conflict with the GPEI's biomedical model of illness. *Shan-Inna*, the Hausa term for polio, is believed to be caused by a powerful female spirit (Yahya, 2006). The rituals of traditional healers – a well-respected part of the social fabric of northern Nigerian communities – are the treatment (Stock, 1985; Renne, 2010); many believed simple drops of OPV could not replace such powerful rituals (Yahya, 2006).

Similarly, religious beliefs surrounding OPV complicated the issue. Abdullahi dan Fodio, the revered nineteenth-century religious scholar and brother of the founder of the Sokoto Caliphate in northern Nigeria, raised queries over accepting healthcare from non-Muslim providers (Abdulla, 1997). With the introduction of biomedicine under colonialism, hospitals were viewed as objects of Western power and attracted suspicion (Good, 1991; Renne, 2008).

Equally difficult to combat was the perception that health and illness are given from Allah: ‘to complain of ill-health is to lodge a complaint against Him – which is almost unthinkable’ (Last, 2005, p. 4). Despite religious obligations stated in the Quran to protect life, local attitudes to immunisation were conflicted: ‘how does immunising a child help if health is preordained?’ (Yahya, 2006). Almsgiving, known as *zakat*, is institutionalised in Islam and the role of lame beggars well defined in Hausa society, to the extent that begging was viewed as a profession (Renne, 2010).

It is imperative to examine local morality when investigating vaccine rumours. Kaler (2004) uses the concept of the ‘moral lens’ to understand hostility to outside medical interventions, noting that such interventions do not arrive in communities as ‘neutral, value-free objects’ (p. 105); rather, they must be understood in local context. The moral lens of northern Nigerians in 2003 was focused clearly on the neo-imperialist threat to African Muslims posed by OPV.

### Politics

Domestic political events around this time strengthened perceived cultural divisions between Nigerians. In April 2003, Obasanjo was re-elected President over the northern, Muslim candidate Muhammadu Buhari. Though unofficial zoning of the presidency (rotating office through regions) is customary and therefore Obasanjo's re-election was expected (Ibrahim, 2009), many in the north resented the reforms of his first tenure, claiming they showed favouritism to the Yoruba-dominated south-western states (Suberu & Diamond, 2002). The 2002 Sharia dispute did little to improve his popularity in the north (Mustapha, 2003).

April 2003 also saw elections for state Governors who, in a federal system like Nigeria, hold considerable political power. In Kano, Ibrahim Shekarau replaced the incumbent from Obasanjo's party, partly due to support from Buhari. Shekarau was particularly eager to assert his new power and resist ‘interference’ from the Federal government. According to Kaufmann and Feldbaum (2009), ‘it was in his interest to make things difficult for President Obasanjo’ (p. 1094). Others agree that ‘the situation got hijacked’ (Barbara Reynolds, UNICEF, quoted in Dugger & McNeil, 2006). One WHO official claimed ‘polio vaccinations just became a pawn in their larger strategy to secure more resources from the Federal Government’ (personal communication, April 2011).

The GPEI in Nigeria, which had a large number of well-paid jobs, was especially susceptible to politicisation. Lucrative positions were under the control of the Ministry of Health and National Programme on Immunisation, both led by southern Yorubas (Renne, 2010). In Nigeria, kinship and patron-clientism are ingrained into government bureaucratic structures, including health (Smith, 2003; Willott, 2011), and the resulting patronage alienated northern communities (Yahya, 2006).

### Society

Religious and political divisions were exacerbated by social disadvantages in many northern states; poverty is much higher than in other areas of Nigeria (Okunmadewa et al., 2005) and service provision is poor (Mustapha, 2006). Adult literacy amongst women in Kano is just 20% according to the Department for International Development (DFID, 2009) and education for women is especially neglected (though many have some Islamic education). The resulting population of less-educated parents lacked the ability to critically appraise rumour credibility; such uncritical groups are often more likely to unquestioningly believe and pass on rumours (Buckner, 1965).

The health system was also neglected. Routine immunisation rates in northern Nigeria were amongst the lowest in the world, with just 4% of children receiving a full course of immunisations in 2003 (FBA Health Systems Analysts, 2005). The decline in immunisation coverage was indicative of a greater decline in primary health care (Bradford & Strozier, 2010; PRRINN-MNCH, 2009). Partly as a result of a lack of education, 70% of parents in Kano believed that polio immunisation protected against all childhood disease including malaria and vomiting (Babalola & Aina, 2004). When these expectations were inevitably not met, confidence declined.

That OPV was offered free amidst more pressing health concerns mystified many and this dichotomy of priorities understandably created resentment (Yahya, 2006). Datti Ahmed implied a lack of care on the part of the GPEI: ‘If the donor agencies are sincere, they should help the country in combating the more dangerous killer diseases’ (Raufu, 2003b, p. 1).

Historical experiences of the pharmaceutical industry in Kano also undermined public trust in Western medical interventions. During a bacterial meningitis outbreak in 1996 the American pharmaceutical company Pfizer conducted a clinical trial in Kano allegedly without licence, ethical approval or informed consent and several children died (Frishman, 2009; Nyike, 2003). To a Nigerian panel of medical experts it was a ‘clear case of exploitation of the ignorant’ (Stevens, 2006, p. 1). Locals apportioned blame broadly: one northern Nigerian stated ‘we cannot trust the white man or our Federal government because many years ago they were in partnership when they brought medicine to poison our people’ (quoted in Yahya, 2006, p. 30). A court case against Pfizer began in April 2003, bringing the controversy back into the public eye (Raufu, 2003a). Dr Ahmed explicitly referenced this when he called for immunisation campaigns to be suspended (Raufu, 2003b).

There was also a more general suspicion of ‘western’ interventions in some parts of northern Nigeria, including Kano. The now infamous Islamist militant group *Boko Haram* (meaning ‘western education is forbidden’) was founded in 2002, one year before the boycott (Onuoha, 2010). Other authors document a violent rejection of all things Western, including biomedicine. Lubeck (1985) and Renne (1996), writing long before the OPV boycott, warned that this might extend to UN programmes in the future. Sadly Renne's predictions were proved correct when, in August 2011, Boko Haram targeted the UN building in Abuja and killed 21 people.

The GPEI was a natural scapegoat for anti-Western sentiments at the time: rumours generally tend to target high-profile organisations (Fine, 1980). Where routine immunisation is poor, such as in northern Nigeria, the GPEI relied on mass campaigns and door-to-door ‘mop up’ campaigns to ensure children receive vaccinations (De Quadros et al., 1991), making the GPEI the most visible local health programme. Taylor (2003) noted that as awareness of polio campaigns increased, GPEI became a target for disruptive groups. In May 2003 an ‘unprecedented tactical shift’ in GPEI policy cited Nigeria as a key country to target (GPEI, 2003), which increased the visibility of the initiative.

These local factors were integral in stimulating and sustaining these rumours at a grass-roots level. Whilst an examination of the international negotiations around the boycott is invaluable, such study must be set within the context of local politics, which, in northern Nigeria, is heavily built around individuals (Joseph, 1983).

## The individuals

Whilst societal factors provided a fertile ground for rumours to spread, the ‘individual-centric’ politics in Nigeria (Ihonvbere, 1994) was fundamental to inflaming the disputes around OPV; the eventual prompts of the boycott were the public views of some very high-profile local leaders. Here, we analyse four key individuals and groups: Dr Datti Ahmed – president of the SCSN; Ibrahim Shekarau – newly elected Governor of Kano; Professor Haruna Kaita – Dean of the Faculty of Pharmaceutical Sciences at Ahmadu Bello University in Zaria; and several Emirs (traditional and religious leaders) of northern Nigeria.

A body of work from sociology is dedicated to understanding the influence of individuals within societies. Rogers's (1962) seminal work *Diffusion of innovations* identified key individuals within a population as ‘innovators’ and Venkatraman (1989) stratified populations into ‘opinion leaders’ and ‘adopters’. Rosen (2000) built on these definitions to suggest four types of network hubs that confer authority: ‘Regular hubs’ influence small numbers of closely related individuals. ‘Megahubs’ are professional opinion leaders like journalists and politicians who are expected to air their views in public and ‘expert hubs’ are specialised opinion leaders (such as scientists) who are looked to for their authority on certain issues. How the GPEI engaged with such Nigerian ‘hubs’ has not been fully examined in the literature, and provides both encouraging and salutary lessons for responses to similar issues in the future.

It is also possible to conceptualise these hubs as a ‘counter-epistemic community’, in forming a group of scientific, religious and political experts at odds with the ‘dominant epistemic community’, in this case the prevailing view of the global health community (Youde, 2005). It is important to note that despite – and partly because – of this opposition, their views had considerable traction with northern Nigerian communities.

### Dr Datti Ahmed

The official prompts of the boycott were the views of Dr Datti Ahmed, a Kano-based physician and head of the SCSN. In July 2003 he called for OPV to be suspended, claiming to have evidence that it was contaminated with ‘anti-fertility substances’ (Raufu, 2003b).

As a physician Dr Ahmed's views on immunisation were well respected in Kano. Such ‘expert hubs’ have played central roles in previous vaccination rumour episodes; the MMR controversy in the UK hinged on one physician's outspoken opinion (Triggle, 2010) and Sweden experienced a dramatic increase in pertussis cases after one expert publicly questioned the need for vaccination (UNICEF, 2005).

As head of the SCSN, Dr Ahmed found his views heeded by local religious leaders (‘regular hubs’) – imams, traditional healers and Islamic teachers who all played a role in sustaining the boycott (BBC, 2002; Yahya, 2007).

### Ibrahim Shekarau

The new governor of Kano, Ibrahim Shekarau, had benefited from the support of the SCSN during the recent election campaigns and also felt obliged to listen to Dr Ahmed's demands. Shekarau publicly stated that ‘it is the lesser of two evils to sacrifice 2, 3, 4, 5, even 10 children [to polio] than allow hundreds of thousands or possibly millions of girl children likely to be rendered infertile’ (Associated Press, 2004a, p. 2).

Although the GPEI was initially hampered by WHO's mandate allowing contact solely with Federal governments, successful resolution required directly speaking to the key individuals involved. David Heymann – the then representative of the Director General for Polio Eradication – challenged convention by contacting Shekarau directly, persuading him to establish a paediatric committee to discuss polio vaccination and eventually convincing him to publicly vaccinate his own daughter (GPEI, 2009; Heymann, personal communication, 2011).

### Emirs

Traditional leaders, another ‘megahub’, had their own concerns. The live OPV rarely causes a vaccine-associated paralytic poliomyelitis or even mutates into a virulent circulating vaccine-derived poliovirus (Kew et al., 2004). Changing epidemiological patterns meant many polio-free countries had switched to the more expensive Inactivated Polio Vaccine (IPV). This opened the door to accusations of double standards in public health (Nathanson & Fine, 2002), and the Emir of Dutse Alhaji Nuhu Muhammadu Sanusi publically wondered, ‘if oral vaccine is not good for American children, why should it be used for Nigerian children?’ (Emir of Dutse, 2003, p. 1).

The GPEI is, by definition, a global effort and often relied upon a directive, top-down approach – successful in the Americas but more troublesome where health infrastructure was less developed (Godlee, 1995). This alienated traditional leadership structures that felt the GPEI was not following ‘the correct norms … the African way of doing things’ (Emir of Kazaure, in Rosenstein & Garrett, 2006, p. 3). UNICEF was responsible for global vaccine procurement and the Federal government was tasked with coordinating immunisation campaigns; partners who inspired little public trust in northern Nigerian communities (Renne, 1996). Later, during testing of the vaccine, this close relationship between the Federal government and international agencies allowed Dr Ahmed to dismiss results from all ‘interested parties like UNICEF’ (Associated Press, 2003).

It was therefore necessary for the GPEI to rapidly establish local ownership to enable immunisations to resume. Politicians were thus given the autonomy to adapt the initiative to the local context, and purchase vaccines independently from the predominantly Islamic country of Indonesia. It was this political freedom that allowed Shekarau and the Emirs to resume vaccinations without losing political capital.

In an effort to engage directly with these leaders, Kofi Annan, the then UN Secretary General, recognised the specific power of influential individuals in Nigeria and personally sent his highest African advisor to Nigeria. Ibrahim Gambari was chosen for his unique family background (his father a northern Nigerian Muslim Emir and mother from the south), which enabled him – in the words of Obasanjo – ‘to get to where I find it difficult to get to’ (quoted in Kaufmann & Feldbaum, 2009, p. 1094). He made an ideal negotiator, capable of discussing directly with key network hubs such as Datti Ahmed, Ibrahim Shekarau, Muhammadu Buhari, local Emirs and the Federal government. This high-profile visit sought to reassure leaders like the Emir of Kazaure who had been outraged by the GPEI's flouting of local hierarchy (Agence France Presse, 2003; Rosenstein & Garrett, 2006).

A special effort was also made to engage with other key individuals. Muhammadu Maccido, the Sultan of Sokoto, the highest ranking traditional and spiritual leader in northern Nigeria, had refused to comment on polio vaccination early in the boycott. After targeted meetings and engagement from GPEI representatives, the Sultan publicly declared OPV safe in March 2004 and personally led vaccination drives (UNICEF, 2004).

To counter the accusation that polio vaccination was an attack on the Islamic world, the Organisation of the Islamic Conference declared support for the initiative in October 2003, soon followed by the Arab League and African Union (Fleshman, 2004b; News24, 2004). Saudi Arabia considered compulsory vaccination for all Kano travellers on *Hajj* to Mecca, which cast further doubt on the assertion that OPV was part of a religious crusade against Muslims. Fatwas were even issued through religious networks advocating vaccination (Kaufmann & Feldbaum, 2009).

Whilst skilful and sensitive negotiations eventually persuaded religious and political leaders to support vaccination, engagement with the Nigerian scientific community was less successful.

### Professor Haruna Kaita

In an initial response to the claims of contaminated vaccines, the Federal government established a technical committee to test OPV and prove its safety. The SCSN and other Islamic groups felt excluded and rejected by the committee. In response, the Federal government included prominent Muslim figures whilst still neglecting members of the SCSN. The SCSN refused to acknowledge the compromise (Chen, 2004). The stalemate eventually achieved partial resolution with separate research groups from the Federal government, religious organisations and Kano authorities performing separate tests in separate countries – South Africa, India and Indonesia (Jegede, 2007).

This initial confusion laid the foundations for further controversy: national government testing declared the vaccines safe and the Federal government pledged to resume vaccinations, but this was quickly contradicted at the state level (Associated Press, 2004b; Fleshman, 2004a). Professor Haruna Kaita, who had chaired the team testing the vaccine in India, announced he had found traces of oestrogen in the vaccine and, warning that it can act as an infertility agent (Weekly Trust, 2004), declared the vaccine ‘unwholesome, substandard and fake’ (Kaita, 2004, p. 44).

The GPEI attributed this to mild impurities in the water used in the testing process, adding that even if oestrogen was present at the levels claimed it would be ‘absolutely of no health consequence’ (Fleshman, 2004b; News24, 2004). Kaita was adamant, claiming the GPEI argument was ‘absurd… very disturbing and ridiculous’ (Weekly Trust, 2004), a view supported by other researchers (Yahya, 2006). In his view, ‘the polio authorities mismanaged a simple issue’ by refusing to engage scientifically with northern Nigerians, instead ‘conjuring religious or political issues’ in an ‘unwitting media war’ against their legitimate concerns (Kaita, personal communication, 2011). The research community of northern Nigeria clearly did not feel listened to, and the resulting confusion delayed the prompt resolution of the boycott.

## Conclusion

The rumours that caused the boycotts in northern Nigeria had traction because of a number of contextual circumstances unique to northern Nigeria; socio-economic marginalisation, dichotomous priorities, historic precedents of bad experiences and contemporary conflicts all played a part in undermining trust in GPEI. The rancorous situation following presidential elections in 2003 converged with other prompters to provide a culture for the rumour to grow and spread at a grass-roots level.

Influential, high-profile individuals supported and propagated these rumours and embodied the broader political, religious and societal reasons for refusing vaccination. International policies aimed at ending the OPV boycott were well constructed and ultimately successful. Whilst the diplomatic response was a successful case study of global health diplomacy, the GPEI had more mixed success in responding to key individuals and this delayed an effective resolution. Some major successes like winning the support of the Sultan of Sokoto and eventually Governor Shekarau were tarnished by the continued discontent of Dr Ahmed and Professor Kaita.

In the year that Kano continued its boycott, polio incidence in Nigeria increased and the Nigerian virus spread across the world (Aylward & Heymann, 2005). Prompt recognition of the specific concerns of high-profile individuals, and the specific concerns of the populations they represent, would enable more rapidly targeted engagement responses to any future crises. Whilst detailed recommendations of exactly how individual and community involvement can be translated into programmatic changes are beyond the scope of this article, the Independent Monitoring Board for polio eradication (IMB) suggests that locally based risk assessments to get a ‘worms-eye view’ (IMB, 2011, p. 3) should be routinely factored into vaccination programmes, whilst Larson and Heymann (2010) acknowledge that the most effective prevention for vaccination rumours lies in the long-term building of public trust. The GPEI continues to build upon this, engaging with groups such as the Northern Traditional Leaders’ Committee on Health Care Delivery’ (Adeyemi, 2010).

With the growing presence of *Boko Haram* in northern Nigeria, the news of a CIA vaccine plot in another Islamic country that broke in July 2011, violence towards vaccinators in some parts of Pakistan and the intensification of the GPEI with the launch of their ‘endgame’ strategy, attention needs to be paid now to strengthen engagement with local communities, in Nigeria and elsewhere.

As we enter what are hopefully the final years of polio eradication, a decade after the northern Nigeria OPV boycott, another crisis such as the one in 2003 could derail hard-won successes and undermine fragile confidence in the polio eradication effort. Important lessons about responding to vaccine rumours were learnt the hard way in northern Nigeria in 2003, with a resulting delay in resolution of the crisis. The response to future crises must build upon the diplomatic success of the 2003 boycott, whilst recognising the importance of community values and the power of individuals. This analysis identifies the convergence of events, individuals and signals that led to the Nigerian boycott, an awareness of which may pre-empt and mitigate future crises.

## References

[R1] Abdulla I. (1997). Islam, medicine, and practitioners in Northern Nigeria.

[R2] Adeyemi K. (2010, February 12). Northern traditional leaders seek eradication of polio. The Nation.

[R3] Afigbo A., Ekeh P., Osaghae E. (1989). Federal character: Its meaning and history. Federal character and federalism in Nigeria.

[R4] Agence France Presse. (2003, October 29). Polio: Anti-African conspiracy. News 24..

[R5] Associated Press. (2003, October 29). Nigeria orders polio vaccine tests.

[R6] Associated Press. (2004a, February 26). Nigerian leader defends polio vaccine boycott. IOL News..

[R7] Associated Press. (2004b, July 31). Nigerian state resumes polio vaccination. USA Today..

[R8] Aylward R. B., Heymann D. L. (2005). Can we capitalize on the virtues of vaccines? Insights from the polio eradication initiative. American Journal of Public Health.

[R9] Aylward R. B., Tangerman R. (2011). The Global Polio Eradication Initiative: Lessons learned and prospect for success. Vaccine.

[R10] Babalola S., Aina O. (2004). Community and systemic factors affecting the uptake of immunisation in Nigeria: A qualitative study in five states (DFID-Nigeria Report).

[R11] BBC. (2002, July 27). Nigeria Muslims oppose polio vaccination..

[R12] Bego A. (2003, November 19). Polio vaccine crisis, not peculiar to Nigeria – WHO Director.

[R13] Bradford C., Strozier M. (2010). PRRINN-MNCH annual review executive summary.

[R14] Buckner H. (1965). A theory of rumour transmission. Public Opinion Quarterly.

[R15] Chen C. (2004). Rebellion against the polio vaccine in Nigeria: Implications for humanitarian policy. African Health Sciences.

[R16] Closser S. (2010). Chasing polio in Pakistan: Why the world's largest public health initiative may.

[R17] Department for International Development. (2009). Nigeria: Country partnership strategy.

[R18] De Quadros C., Andrus J., Olive J., Da Silveira C., Eikhof R., Carrasco P., Pinheiro F. (1991). Eradication of poliomyelitis: Progress in the Americas. The Pediatric Infectious Disease Journal.

[R19] Dugger C., McNeil D. (2006, March 20). Rumor, fear and fatigue hinder final push to end polio. New York Times..

[R20] Emir of Dutse. (2003). Why we refused polio vaccination in Jigawa state [interview transcript]. All Africa.

[R21] FBA Health Systems Analysts. (2005). The state of routine immunization services in Nigeria and reasons for current problems.

[R22] Fine G. A. (1980). The Kentucky fried rat: Legends and modern society. Journal of the Folklore Institute.

[R23] Fleshman M. (2004a). Nigeria dispute endangers global polio drive. Africa Recovery, United Nations.

[R24] Fleshman M. (2004b). UN mediates polio deadlock in Nigeria. Africa Renewal, United Nations.

[R25] Frishman A. (2009). Major reason for Nigerian boycott of polio vaccine. Health Affairs.

[R26] Global Polio Eradication Initiative. (2003, May 13). Changing epidemiology of polio prompts tactical shift in world's largest public health initiative. Press Release..

[R27] Global Polio Eradication Initiative. (2009). Bill Gates visits Nigeria.

[R28] Global Polio Eradication Initiative. (2013). Media Room.

[R29] Godlee F. (1995). The World Health Organization: WHO special programmes: Undermining from above. British Medical Journal.

[R30] Good C. (1991). Pioneer medical missions in colonial Africa. Social Science and Medicine.

[R31] Harnischfeger J. (2004). Sharia and control over territory: Conflict between “settlers” and “indigenes” in Nigeria. African Affairs.

[R32] Heymann D. L., Sutter R., Aylward R. B. (2006). A vision of a world with polio: The OPV cessation strategy. Biologicals.

[R33] Ibrahim J. (2009). Nigeria's 2007 elections: The fitful path to democratic citizenship.

[R34] Ihonvbere J. (1994). Nigeria: The politics of adjustment and democracy.

[R35] Independent Monitoring Board for Polio Eradication. (2011). April 2011 report.

[R36] IRIN. (2004). Two more northern states ban polio vaccination.

[R37] Jegede A. (2007). What led to the Nigerian boycott of the polio vaccination campaign?. PLoS Medicine.

[R38] Joseph R. (1983). Class, state and prebendal politics in Nigeria. The Journal of Commonwealth and Comparative Politics.

[R39] Kaita H. (2004). Polio immunisation and child survival.

[R40] Kaler A. (2004). The moral lens of population control: Condoms and controversies in Southern Malawi. Studies in Family Planning.

[R41] Kaufmann J., Feldbaum H. (2009). Diplomacy and the polio immunization boycott in Northern Nigeria. Health Affairs.

[R42] Kew O., Wright P., Agol V., Delpeyroux F., Shimizu H., Nathanson N., Pallansch M. (2004). Circulating vaccine-derived polioviruses: Current state of knowledge. Bulletin of the World Health Organization.

[R43] Kimmel A. (2004). Rumours and rumour control.

[R44] Larson H., Ghinai I. (2011). Lessons from polio eradication. Nature.

[R45] Larson H., Heymann D. L. (2010). Public health response to influenza A(H1N1) as an opportunity to build public trust. Journal of the American Medical Association.

[R46] Larson H., Smith D., Paterson P., Cumming M., Eckersberger E., Freifeld C, Madoff L. (2013). Measuring vaccine confidence: Analysis of data obtained by a media surveillance system used to analyse public concerns about vaccines. Lancet Infectious Diseases.

[R47] Last M., Falola T. (2005). Religion and healing in Hausaland. Christianity and social change in Africa: Essays in honour of J. D. Y. Peel.

[R48] Last M. (2008). The search for security in Muslim Northern Nigeria. Africa: The Journal of the International African Institute.

[R49] Lubeck P. M. (1985). Islamic protest under semi-industrial capitalism: ‘Yan Tatsine Explained’. Africa.

[R50] Murphy J. (2004, January 4). Distrust of US foils effort to stop crippling disease. The Baltimore Sun..

[R51] Mustapha A., Samatar A., Samatar A. (2002). Coping with diversity: The Nigerian state in historical perspective. The African state: Reconsiderations.

[R52] Mustapha A., Burman B., Eyoh D., Kymlicka W. (2003). Ethnicity and politics of democratisation in Nigeria. Ethnicity and democracy in Africa.

[R53] Mustapha A. (2006, November 16). Ethnic structure, inequality and governance of the public sector in Nigeria..

[R54] Nathanson N., Fine P. (2002). Poliomyelitis eradication: A dangerous endgame. Science: Virology.

[R55] News24 (2004). Vaccine boycott spreads polio.

[R56] Nnoli O. (1978). Ethnic politics in Nigeria.

[R57] Nyike A. (2003). The Trovan trial case study: After profits or to save lives?.

[R58] Okunmadewa F., Olaniya O., Yusuff A., Bankole A., Oyeranti O., Omonona B., Olayiwola K. (2005). Human capital, institutions and poverty in rural Nigeria.

[R59] Onuoha F. (2010). The Islamist challenge: Nigeria's Boko Haram crisis explained. African Security Review.

[R60] PRRINN-MNCH. (2009). Baseline studies 2009 summary report.

[R61] Raufu A. (2002). Polio cases rise in Nigeria as vaccine is shunned for fear of AIDS. British Medical Journal.

[R62] Raufu A. (2003a). Nigerians in drug trial take their case to US Court. British Medical Journal.

[R63] Raufu A. (2003b). Polio vaccine plans may run into problems in Nigeria. British Medical Journal.

[R64] Renne E. P. (1996). Perceptions of population policy, development, and family planning programs in Northern Nigeria. Studies in Family Planning.

[R65] Renne E. P. (2008). Islam and immunisation in Northern Nigeria. Protesting polio and the ethics of eradication in Northern Nigeria.

[R66] Renne E. P. (2010). The politics of polio in Northern Nigeria.

[R67] Rogers E. M. (1962). Diffusion of innovations.

[R68] Rosen E. (2000). The anatomy of a buzz: How to create word of mouth marketing.

[R69] Rosenstein S., Garrett L. (2006). Polio's return, a WHO-done-it. The American Interest.

[R70] Smith D. (2003). Patronage, per diems and the “workshop mentality”: The practice of family planning programs in Southeastern Nigeria. World Development.

[R71] Stevens J. (2006, May 7). Panel faults Pfizer in '96 clinical trial in Nigeria. Washington Post.

[R72] Stock R. (1985). Health care for some: A Nigerian study of who gets what, where and why. International Journal of Health Sciences.

[R73] Suberu R., Diamond L., Reynolds A. (2002). Institutional design, ethnic conflict-management and democracy in Nigeria. The architecture of democracy.

[R74] Taylor S. (2003). Social mobilisation and communication for polio eradication: Documentation in Nigeria, India, and Pakistan..

[R75] Taylor S., Shimp L. (2010). Using data to guide action in polio health communications: Experience from the Polio Eradication Initiative. Journal of Health Communication.

[R76] Thacker N., Shendurnikar N. (2004). Current status of polio eradication and future prospects. Indian Journal of Pediatrics.

[R77] Triggle N. (2010, January 28). Wakefield and autism: The story that will not go away. BBC News.

[R78] Ukiwo U. (2003). Politics, ethno-religious conflicts and democratic consolidation in Nigeria. Journal of Modern African Studies.

[R79] UNICEF. (2001). Combating antivaccination rumours: Lessons learnt from case studies in East Africa..

[R80] UNICEF. (2004). Traditional chiefs from Niger and Nigeria join together to fight polio.

[R81] UNICEF. (2005). Building trust and responding to adverse events following immunisation in South Asia: Using strategic communication..

[R82] UNICEF. (2012). Root causes of refusals revealed through DR Congo study.

[R83] Venkatraman M. (1989). Opinion leaders, adopters and communicative adopters: A role analysis. Psychology & Marketing.

[R84] Walsh D. (2007, February 14). Polio cases jump in Pakistan as clerics declare vaccination an American plot. The Guardian.

[R85] Weekly Trust. (2004). Oral polio vaccine is junk: “Our polio test was conclusive” – Dr Haruna Kaita.

[R86] Willott C. (2011). Get to the bridge and I will help you to cross”: Merit, personal connections and money in access to Nigerian higher education. Africa Spectrum.

[R87] Yahya M. (2006). Polio vaccines – difficult to swallow. The story of controversy in Northern Nigeria.

[R88] Yahya M. (2007). Polio vaccines – “no thank you!” Barriers to polio eradication in Northern Nigeria. African Affairs.

[R89] Youde J. (2005, 1–5 March). South Africa, AIDS, and the development of a counter-epistemic community..

